# Diagnosis of Tuberculosis in the Wild Boar (*Sus scrofa*): A Comparison of Methods Applicable to Hunter-Harvested Animals

**DOI:** 10.1371/journal.pone.0012663

**Published:** 2010-09-10

**Authors:** Nuno Santos, Margarida Geraldes, Andreia Afonso, Virgílio Almeida, Margarida Correia-Neves

**Affiliations:** 1 Life and Health Sciences Research Institute (ICVS), School of Health Sciences, University of Minho, Braga, Portugal; 2 Pygargus Lda, Póvoa Lanhoso, Portugal; 3 Centro de Investigação Interdisciplinar em Sanidade Animal – Faculdade de Medicina Veterinária (CIISA/FMV), TULisbon, Polo Universitário do Alto da Ajuda, Lisbon, Portugal; 4 National Institute of Biological Resources, Veterinary Research National Laboratory (LNIV), Vairão, Portugal; MRC Laboratories, Gambia

## Abstract

**Background:**

To obtain robust epidemiological information regarding tuberculosis (TB) in wildlife species, appropriate diagnostic methods need to be used. Wild boar (*Sus scrofa*) recently emerged as a major maintenance host for TB in some European countries. Nevertheless, no data is available to evaluate TB *post-mortem* diagnostic methods in hunter-harvested wild boar.

**Methodology/Principal Findings:**

Six different diagnostic methods for TB were evaluated in parallel in 167 hunter-harvested wild boar. Compared to bacteriological culture, estimates of sensitivity of histopathology was 77.8%, gross pathology 72.2%, PCR for the MPB70 gene 66.7%, detection of acid-fast bacilli (AFB) in tissue contact smears 55.6% and in histopathology slides 16.7% (estimated specificity was 96.7%, 100%, 100%, 94.4% and 100%, respectively). Combining gross pathology with stained smears in parallel increased estimated sensitivity to 94.4% (94.4% specificity). Four probable bacteriological culture false-negative animals were identified by Discriminant Function Analysis. Recalculating the parameters considering these animals as infected generated estimated values for sensitivity of bacteriology and histopathology of 81.8%, gross pathology 72.7%, PCR for the MPB70 gene 63.6%, detection of AFB in tissue contact smears 54.5% and in histopathology slides 13.6% (estimated specificity was 100% for gross pathology, PCR, bacteriology and detection of AFB in histopathology slides, 96.7% for histopathology and 94.4% for stained smears).

**Conclusions/Significance:**

These results show that surveys for TB in wild boar based exclusively on gross pathology considerably underestimate prevalence, while combination of tests in parallel much improves sensitivity and negative predictive values. This finding should thus be considered when planning future surveys and game meat inspection schemes. Although bacteriological culture is the reference test for TB diagnosis, it can generate false-negative results and this should be considered when interpreting data.

## Introduction

*Mycobacterium bovis* (*M. bovis*) has the widest host range of any member of the *Mycobacterium tuberculosis* complex (MTC), infecting many species of wild and domestic mammals and also man [1], [2], and causes tuberculosis (TB).

TB occurs in domestic animals worldwide, although several countries successfully eradicated TB in cattle through test and slaughter programs and abattoir surveillance. In some other countries (e.g. United Kingdom, USA, New Zealand) the disease is re-emerging. These later countries have in common the existence of wildlife reservoir species [1].

Several wildlife species have been reported as maintenance hosts for *M. bovis*, including ungulates, carnivores and marsupials. Regarding free-ranging suids, TB was reported in feral pigs (*Sus scrofa*) in Oceania and Pacific islands, warthog (*Phacochoerus aethiopicus*) in Africa and wild boar (*Sus scrofa*) in Europe [1], [3]. There is evidence that the wild boar is a maintenance host for *M. bovis* in the Iberian Peninsula [4], where wildlife TB is re-emerging [5], [6].

Available *post-mortem* tests for TB include gross pathology, examination of Ziehl-Neelsen (ZN) stained contact smears of tissues for acid-fast bacilli (AFB), histopathology aimed at detecting AFB or tuberculosis-like lesions (TBL), PCR and bacteriological culture [1]. Although expensive and extremely time-consuming, bacteriological culture is considered the reference test for the diagnosis of TB, as most other techniques lack sensitivity and/or specificity [1], [7], [8].

Nevertheless, bacteriological culture can give rise to false negative results [8], which is particularly problematic when other diagnostic methods are being evaluated. In fact, the reference test is assumed to have 100% sensitivity, which can be unrealistic and generate false parameters for the other diagnostic methods being evaluated. It is therefore essential to estimate the true sensitivity of bacteriological culture when evaluating other diagnostic methods.

As the financial resources needed for performing bacteriological culture on a large number of samples are rarely available and because this technique is extremely time-consuming, most surveys use other methods (usually gross pathology) as screening tests, and only perform culture for lesion-positive animals, sometimes as pooled samples (e.g., [6], [9], [10]). In order to calculate the real prevalence of TB from surveys based on other diagnostic tests, it is imperative to estimate the sensitivity, specificity, positive and negative predictive values (PPV and NPV, respectively) of these tests [7]. This evaluation is seldom done for wildlife species due to the intrinsic difficulties of working with these species, including difficult access to animals and samples and the fact that collection of samples usually takes place in remote locations, where conditions often are not the most appropriate [11]. When dealing with hunter-harvested animals, bullet wounds, partial consumption of viscera by dogs and tissue contamination with feces or soil often preclude obtaining tissues in good conditions.

Interestingly, no data is available on diagnostic tests comparison for wild boar TB. Published surveys on wild boar TB (e.g., [5], [10]) rely mostly on culturing only animals with visible macroscopic lesions, which does not allow the calculation of real prevalence. The aim of this study was to compare *post-mortem* diagnostic tests for TB in hunter-harvested wild boar. By estimating sensitivity, specificity, PPV and NPV for each test, we propose a combination of tests that is best suited for large-scale surveys of TB in this species. We have also estimated the extent of occurrence of false-negatives in the reference test, by Discriminant Function Analysis, in order to correctly evaluate all diagnostic tests.

## Materials and Methods

### Ethics statement

This study didn't involve purposeful killing of animals. Samples were collected from wild boar legally hunted for recreational purposes. No ethical approval was deemed necessary.

### Collection and processing of samples

Samples were collected from submandibular, retropharyngeal, tracheobronchial and mesenteric lymph nodes and also from lung samples from hunter-harvested wild boar during the 2005–2006 and 2006–2007 hunting seasons. Tissues to collect were selected based on the known location of TB lesions in this species [9], [12], [13]. Age and gender were determined as described elsewhere [6]. After evisceration of the carcasses, performed in the field by the hunters or game meat processing companies 2 to 10 h *post-mortem*, biological samples were collected using different sets of equipment for each animal and stored refrigerated separated by tissue type in sterile 40 ml tubes. Gross pathology, tissue contact smears and histopathology protocols were performed within 48 h of sample's collection. Lymph nodes were kept frozen at −20°C until bacteriology and PCR protocols were performed 4 to 7 months later. All tests were performed for every animal included in the study. Gross pathology and examination of tissue smears were performed individually for every tissue collected, while histopathology, PCR and bacteriological culture were performed on pooled tissue samples from the same animal. All tests were carried out blindly.

### Gross pathology

All collected tissues were cut in thin slices (roughly 3 mm wide), and the presence of macroscopic TBL was recorded. Any granulomatous, caseous, purulent, necrotic, calcified or proliferative lesion was classified as TBL, according to previous reports [9], [12], [13].

### Histopathology

For every animal, 1 to 3 pieces of tissue, including those with detected macroscopic TBL (if detected), were immersed and fixed in 10% neutral buffered formalin. Fixed tissues were dehydrated and embedded in paraffin. Approximately 4 µm thick sections were cut and slides were stained by the ZN and hematoxylin-eosin (HE) methods. Whole slides were examined by light microscopy at 40x and 200x amplifications, and the presence of TBL microscopic lesions, as described by others [9], [12], [13], was recorded (histopathology I). Lesions were characterized by the degree of caseous necrosis, calcification/mineralization and fibrotic capsule. For every animal, roughly one-fourth of the ZN-stained slide was observed at 1,000x amplification and the presence of AFB was recorded (histopathology II).

### Stained smears

For every tissue collected, contact smears were prepared in microscopic slides and stained by the ZN method. Each slide was observed across the whole length of the smear at 1,000x amplification for about 12 min and the presence of AFB was recorded.

### Molecular biology

DNA was extracted from tissue homogenates from every animal (see Bacteriology section) by standard phenol-chloroform method, after 2×30 s agitation with 0.1 mm zirconium beads using a Mini Bead-Beater (Biospec, Bartlesville, USA). It was then dissolved in TE buffer and stored at −20°C, after quantification in an UV spectrophotometer (Beckman DU 650, Beckman Coulter, Fullerton, USA). PCR for MTC-specific gene MPB70 was performed by a modification of the method described by Huard *et al*. [14]. Briefly, we used 5 µl of Taq buffer, 300 mM each dNTP, 1.5 mM MgCl_2_, 2.5 U Taq polymerase (Fermentas, Burlington, Canada), 5% DMSO, 1.5 µl of each primer at 20 µM (F: GGC GAT CTG GTG GGC CCG, R: CGC CGG AGG CAT TAG CAC GCT) and 2 µg of DNA, in a final volume of 50 µl. The PCR protocol was: initial denaturation at 94°C for 5 min, 50 cycles at 94°C for 1 min, annealing at 65°C for 1 min and extension at 72°C for 1 min, with a final extension step at 72°C for 10 min. PCR products were visualized after electrophoresis in a 1% agarose gel with ethidium bromide and photographed under UV light (Alpha Imager, Alpha Innotech Corporation, San Leandro, USA). Each sample was tested twice and any positive result was confirmed by repeating the PCR.

A control PCR for a 662 bp fragment of the swine mitochondrial control region between positions 15661 and 601 according to the reference sequence (Genbank accession number AF034253) [15] was performed in each sample. We used 5 µl of Taq buffer, 200 mM each dNTP, 3 mM MgCl_2_, 2.5 U Taq polymerase (Fermentas, Burlington, Canada), 1.5 µl of each primer at 20 µM (F: ACT AAC TCC GCC ATC AGC AC, R: CTG TGT TAG GGC CTT TGA CG) and 1 µg of DNA, in a final volume of 50 µl. This was submitted to the following PCR protocol: initial denaturation at 95°C for 15 min, followed by 30 cycles at 94°C for 30 s, annealing at 60°C for 90 s and extension at 72°C for 90 s, with a final extension step at 72°C for 10 min.

### Bacteriology

The bacteriology protocol was performed in a BSL3 laboratory as previously described [6]. Briefly, for every animal about 3 g of pooled tissues were homogenized in 4 ml sterile water; 400 µl of the homogenate was frozen for later DNA extraction (see Molecular biology section). The remaining homogenate was decontaminated in 35 ml of a 0.75% hexa-decyl-pyridinium chloride solution. After decontamination for 2 h, a tube with Coletsos medium (BioMerieux, Marcy l'Étoile, France) and a Petri dish with Middlebrook 7H11 medium enriched with OADC (Becton Dickinson, Franklin Lakes, USA) were inoculated with sediment/supernatant interface. After decontamination for 18 h, another plate of Middlebrook 7H11 medium enriched with OADC was seeded. Plates and tubes were incubated at 37°C for 10 wk. Any bacterial growth was inoculated onto a plate of Middlebrook 7H11 medium enriched with OADC and also smeared on a microscopic slide and suspended in 400 µl of sterile water, then frozen at −20°C. The smear was ZN-stained and observed for the presence of AFB. DNA was extracted from the suspension by the method described under Molecular biology.

### Identification of isolates

Bacterial isolates were identified by PCR for a panel of selected genes: 16S RNA, IS1081, Rv3120, Rv1510 and IS1245 [14], [16]. For the first four genes the protocol used was the one described by Huard *et al*. [14]. Briefly, 5 µl of Taq buffer (Fermentas, Burlington, Canada), 200 mM each dNTP, 1.5 mM MgCl_2_, 1.25 U Taq polymerase, 5% DMSO, 1 µl of each primer at 20 µM and 1.25 µg of DNA were mixed in a final volume of 50 µl. This mix was submitted to the following PCR protocol: initial denaturation at 94°C for 5 min, 35 cycles at 94°C for 1 min, annealing at 60°C for 1 min and extension at 72°C for 1 min, with a final extension step of 72°C for 10 min. For IS1245 we followed a protocol described previously by others [16], [17]. Briefly, 5 µl of Taq buffer (Fermentas, Burlington, Canada), 1.5 mM MgCl_2_, 1.25 U Taq polymerase and 1.25 µg of DNA were mixed in a final volume of 50 µl. This mix was submitted to the following PCR protocol: 45 cycles at 94°C for 1 min, 65°C for 1 min and 72°C for 1 min, with a final extension step at 72°C for 10 min. PCR products were visualized after electrophoresis in a 1% agarose gel with ethidium bromide and photographed under UV light (Alpha Imager, Alpha Innotech Corporation, San Leandro, USA). This set of genes allowed the identification of isolates as *M. bovis*, *Mycobacterium caprae*, *Mycobacterium microti*, other members of the MTC, *Mycobacterium avium* complex (MAC) and other mycobacteria that were not MTC or MAC [14], [16].

### Definitions

Case - wild boar with *M. bovis* bacteriological isolation; Negative reference animal - wild boar without *M. bovis* isolation and originating from TB-free study areas; Uncertain status animal - wild boar without *M. bovis* isolation originating from study areas where *M. bovis* was isolated in other wild boar or from study areas with <90% confidence of detecting a prevalence of 15%; TB-free study areas - study areas from where no *M. bovis* was isolated and with >90% confidence of detecting a prevalence of 15%;TB-infested study areas - study areas from where *M. bovis* was isolated.

### Statistical analysis

Statistical analysis was performed with Excel 2002 (Microsoft, Redmond, Washington, USA) and SPSS 13.0 (SPSS, Chicago, Illinois, USA) software. For each diagnostic test we determined sensitivity, specificity, PPV and NPV, with confidence intervals, using available software (VassarStats: web site for statistical computation - http://faculty.vassar.edu/lowry/VassarStats.html). For sensitivity estimation all animals under study were considered, for specificity only negative reference animals and for PPV and NPV estimation both cases and negative reference animals were used. In order to detect possible bacteriological culture false-negatives, Discriminant Function Analysis (DFA) was conducted on the following groups: cases; negative reference animals; and uncertain status animals. DFA produced a model of TB status based on results from diagnostic tests other than bacteriological culture, which was then used to classify animals as presumably infected or not.

## Results

Tissue samples were collected from 189 hunter-killed wild boar from 9 study areas (8 areas reported previously [6] and an additional area included in the present study) in south and central Portugal. Animals for which results were lacking for any of the diagnostic methods performed (e.g. no smears, negative control PCR, etc) were not considered for the data analysis. Thus, the sample analyzed included 167 wild boar, composed of 80 females, 28 males and 59 of undetermined sex; 21 animals were juveniles, 31 subadults, 63 adults and the remaining 52 were of undetermined age. We had access to submandibular lymph nodes from 143 animals, retropharyngeal lymph nodes from 107 animals, tracheobronchial lymph nodes from 88 animals, mesenteric lymph nodes from 63 animals and lung portions from 58 animals.

Bacteriological culture from the 167 wild boar resulted in the isolation of *M. bovis* from 18 animals (P = 10.8%, CI*_P_*_95%_ 6.9–16.4%), MAC from 8 and other mycobacteria not belonging to MTC or MAC from 15 animals (Table S1). No other species from the MTC were detected by culture apart from *M. bovis*. The number of wild boar in each subset of the sample was: cases (n = 18); negative reference animals (n = 90); and uncertain status animals (n = 59).

By gross pathology analysis, TBL were detected in 35/59 tissue samples from 18 animals (range: 1–4 tissues with lesions/animal). Considering only the subset cases, positivity rates for the detection of TBL were 10/15 (66,7%) for submandibular lymph nodes, 5/13 (38,5%) for tracheobronchial lymph nodes, 5/14 (35,7%) for retropharyngeal lymph nodes, 2/11 (18,2%) for mesenteric lymph nodes and 1/11 (9,9%) for lung.

**Figure 1:**
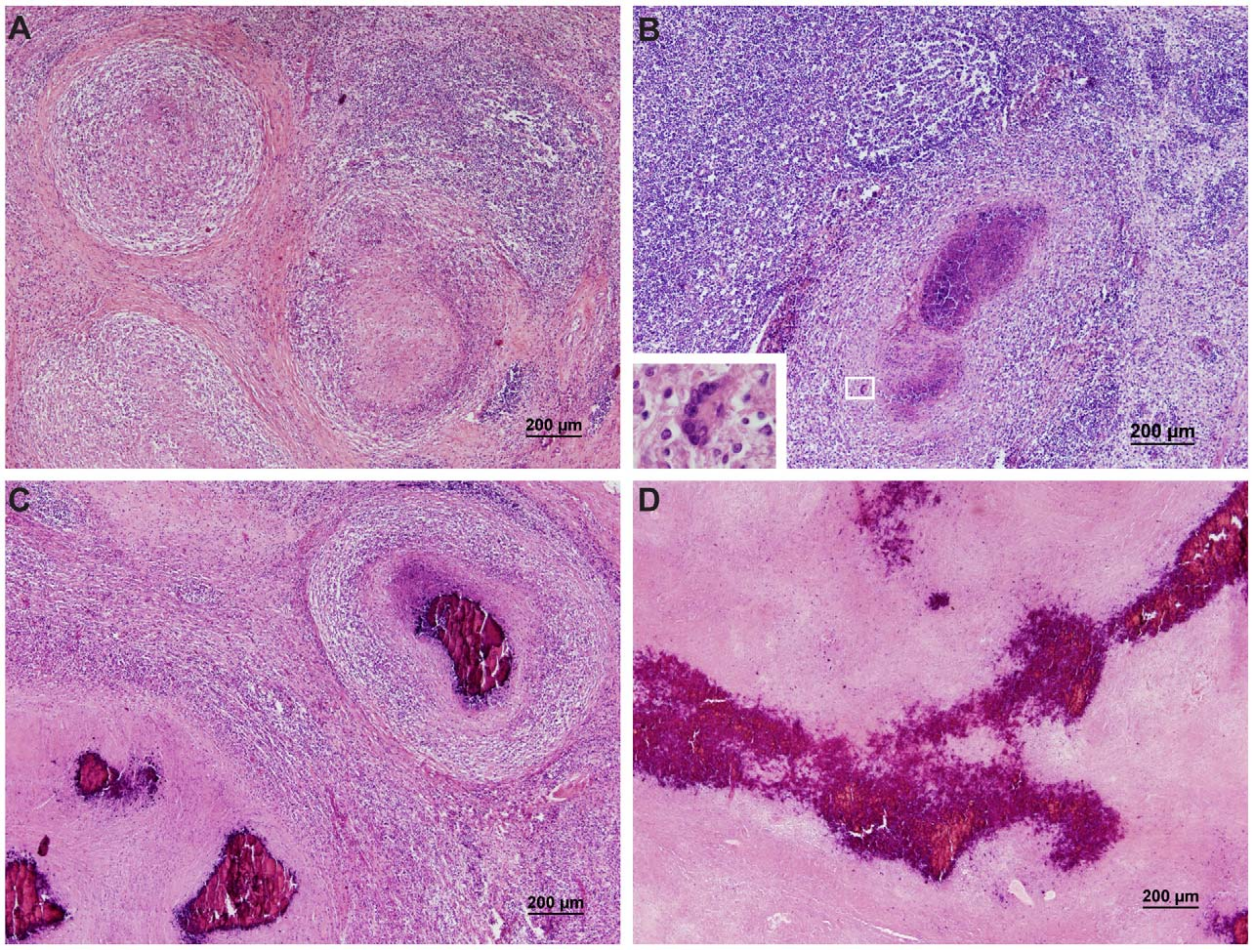
Light Micrograph of lymph node lesions stained with HE. **A**) Granulomatous lesions with necrotic core; **B**) Granulomatous lesions with a central caseous necrosis with light mineralization, surrounded by macrophage-like cells with abundant granular eosinophilic cytoplasm and multinucleate giant cells – Langhans' giant cells (inset); **C**) Two adjacent granulomatous lesions with central mineralized caseous necrosis, bound by macrophage-like cells and fibrosis; **D**) Advanced lesion showing extensive caseous necrotic areas with strong mineralization and fibrosis.

Microscopic TBL (Figure 1) were detected in HE-stained histopathology slides from 22 animals and consisted of granulomatous lesions in 20 animals, characterized by necrotic cores surrounded by accumulations of epithelioid macrophages, macrophage-like cells and sometimes multinucleated giant cells, these later always in small numbers. Additionally dystrophic mineralization in the necrotic areas, with moderate to marked extension were observed in 15 animals. Some of the necrotic calcified and the non-calcified granulomas were limited by connective tissue. Granulomas without caseous necrosis were observed in 2 wild boar.

AFB were detected, always in small numbers, in ZN-stained histopathology slides from 4 animals. AFB were detected in ZN-stained tissue contact smears from 19 animals.

The presence of *M. bovis* DNA (MPB70 gene) was detected by PCR in tissue homogenates from 15 animals.

Estimated sensitivity, specificity, negative and positive predictive values for each test other than bacteriological culture are listed in Table 1.

**Table 1:** Estimates of sensitivity, specificity, positive and negative predictive value with 95% confidence interval for each of the diagnostic tests, compared to bacteriological culture.

Diagnostic test	Sensitivity		Specificity		PPV		NPV	
	%	CI*_P_*_95%_	%	CI*_P_*_95%_	%	CI*_P_*_95%_	%	CI*_P_*_95%_
Gross pathology	72.2	46.4–89.3	100	94.9–100	100	71.7–100	94.7	87.6–98.0
Histopathology I	77.8	51.9–92.6	96.7	89.9–99.1	82.4	55.8–95.3	95.6	88.5–98.6
Histopathology II	16.7	4.4–42.3	100	94.9–100	100	31.0–100	85.7	77.2–91.5
Stained smears	55.6	31.3–77.6	94.4	86.9–97.9	66.7	38.7–87.0	91.4	83.3–95.9
PCR MPB70	66.7	41.2–85.6	100	94.9–100	100	69.9–100	93.8	86.4–97.4

Case definition: wild boar with *M. bovis* bacteriological isolation. Sample used for the calculation of each parameters were: all animals under study (n = 167) for sensitivity, negative reference animals (n = 90) for specificity, cases and negative reference animals (n = 108) for PPV and NPV. Prevalence for PPV and NPV calculation was 16.7%.

The low values for estimated sensitivity obtained prompted us to investigate if the combination of tests in parallel would improve this parameter. Only combinations which significantly improved sensitivity are listed (Table 2).

**Table 2:** Estimates of sensitivity, specificity, positive and negative predictive value with 95% confidence interval for selected combinations of diagnostic tests, compared to bacteriological culture.

Combination of tests in parallel	Sensitivity		Specificity		PPV		NPV	
	%	CI*_P_*_95%_	%	CI*_P_*_95%_	%	CI*_P_*_95%_	%	CI*_P_*_95%_
Histopathology IxStained smears	100	78.1–100	91.1	82.8–95.8	69.2	48.1–84.9	100	94.4–100
Gross pathology xStained smears	94.4	70.6–99.7	94.4	86.9–97.9	77.3	54.2–91.3	98.8	92.8–99.9
Stained smearsxPCR MPB70	88.9	63.9–98.1	94.4	86.9–97.9	76.2	52.5–90.9	97.7	91.2–99.6
Gross pathologyxPCR MPB70	77.8	51.9–92.6	100	94.9–100	100	73.2–100	95.7	88.8–98.6

Case definition: wild boar with *M. bovis* bacteriological isolation. Sample used for the calculation of each parameters were: all animals under study (n = 167) for sensitivity, negative reference animals (n = 90) for specificity, cases and negative reference animals (n = 108) for PPV and NPV. Prevalence for PPV and NPV calculation was 16.7%.

MAC-infected wild boar were negative for all tests, except 1/8 positive for AFB in stained tissue contact smears. Animals from which mycobacteria other than MTC or MAC were isolated, were negative in the other tests, except for 2/15 that were positive for gross pathology (one of which was later shown to be an actinogranuloma), 1/15 positive for AFB in ZN-stained contact smears and 1/15 positive for microscopic TBL lesions (Table S1).

In order to identify possible false-negative bacteriological culture results, we applied DFA to a sub-set of wild boar consisting of cases and negative reference animals. The DFA produced one model with an eigenvalue of 3.628, which explained 100% of the variance in TB status, with the structure matrix listed in Table 3. This model correctly classified 103/108 of cases and negative reference animals. In all 5 animals whose bacteriological results and DFA model classification did not concur (WB13, 127, 128, 130, 171) *M. bovis* was cultured but the model classified them as negative. In 4 of these wild boar no macro or microscopic TBL lesions were identified; besides bacteriology, they were only positive for AFB in stained tissue smears.

**Table 3:** Correlation of each diagnostic test within the DFA model.

Diagnostic test	Correlation within function
Gross pathology	0.773
PCR MPB70	0.678
Histopathology I	0.549
Stained smears	0.361
Histopathology II	0.214

When applied to the uncertain status group, the same model correctly classified 54/59 wild boar as negative. The five animals whose bacteriological results and DFA model classification did not agree have their test profiles presented in Table 4. In 3/5 of these misclassified wild boar (WB15, 120 and 170) we could demonstrate the presence of mycobacteria either by AFB visualization (2 animals) or by PCR (2 animals) (Table 4). WB172 had macro and microscopic TBL but the presence of mycobacteria could not be demonstrated by any of the other methods used. Furthermore, WB17 was only positive for gross pathology and later found by histopathology that those lesions were actinogranulomas, hence WB17 is probably not a bacteriological culture false negative result. Assuming the other 4 wild boar (WB15, 120, 170 and 172) as false-negatives for the bacteriological culture, we adopted a new Case definition: animals classified as positive by the DFA model (excluding WB17). We then recalculated the parameters for each diagnostic test or combination of tests, considering now these as the animals infected by *M. bovis* (Table 5 and Table 6).

**Table 4:** Diagnostic tests profiles for the DFA-misclassified wild boar from the uncertains status group.

ID	Gross pathology	ZN-stained smears	Histopathology I	Histopathology II	PCR MPB70	Bacteriological culture
WB15	-	+	+	-	+	-
WB17	+	-	-	-	-	other mycobacteria
WB120	+	+	+	-	-	other mycobacteria
WB170	+	-	+	-	+	-
WB172	+	-	+	-	-	-

Individual diagnostic test profiles for the 5 wild boar from the uncertain status group (wild boar without *M. bovis* bacteriological isolation from TB-infested areas) whose DFA model classification and bacteriological results differed. “−” negative test result; “+” positive test result; “other mycobacteria” mycobacteria not belonging to MTC or MAC.

**Table 5:** Estimates of sensitivity, specificity, positive and negative predictive value with 95% confidence interval for each of the diagnostic tests, compared to DFA model classification.

Diagnostic test	Sensitivity		Specificity		PPV		NPV	
	%	CI*_P_*_95%_	%	CI*_P_*_95%_	%	CI*_P_*_95%_	%	CI*_P_*_95%_
Gross pathology	72.7	49.6–88.4	100	94.9–100	100	75.9–100	93.8	86.4–97.4
Histopathology I	81.8	59.0–94.0	96.7	89.9–99.1	85.7	62.6–96.2	95.6	88.5–98.6
Histopathology II	13.6	3.6–36.0	100	94.9–100	100	31.0–100	82.6	73.9–88.9
Stained smears	54.5	32.7–74.9	94.4	86.9–97.9	70.6	44.0–88.6	89.5	81.1–94.6
PCR MPB70	63.6	40.8–82.0	100	94.9–100	100	73.2–100	91.8	84.1–96.2
Culture	81.8	59.0–94.0	100	94.9–100	100	78.1–100	95.7	88.8–98.6

Case definition: animals classified as positive by the DFA model (excluding WB17). Sample used for the calculation of each parameters were: all animals under study (n = 167) for sensitivity, negative reference animals (n = 90) for specificity, cases and negative reference animals (n = 112) for PPV and NPV. Prevalence for PPV and NPV calculation was 19.6%.

**Table 6:** Estimates of sensitivity, specificity, positive and negative predictive value with 95% confidence interval for selected combinations of diagnostic tests, compared to DFA model classification.

Combination of tests in parallel	Sensitivity		Specificity		PPV		NPV	
	%	CI*_P_*_95%_	%	CI*_P_*_95%_	%	CI*_P_*_95%_	%	CI*_P_*_95%_
CulturexHistopathology I	100	81.5–100	96.7	89.9–99.1	88.0	67.7–96.8	100	94.7–100
Stained smearsxHistopathology I	100	81.5–100	91.1	82.8–95.8	73.3	53.8–87.0	100	94.4–100
CulturexGross pathology	95.5	75.1–99.8	100	94.9–100	100	80.8–100	98.9	93.2–99.9
Gross pathologyxStained smears	95.5	75.1–99.8	94.4	86.9–97.9	80.8	60.0–92.7	98.8	92.8–99.9
CulturexPCR MPB70	90.9	69.4–98.4	100	94.9–100	100	80.0–100	97.8	91.6–99.6
CulturexSmears	90.9	69.4–98.4	94.4	86.9–97.9	80.0	58.7–92.4	97.7	91.2–99.6
Stained smearsxPCR MPB70	86.4	64.0–96.4	94.4	86.9–97.9	79.2	57.3–92.1	96.6	89.7–99.1
Gross pathologyxPCR MPB70	81.8	59.0–94.0	100	94.9–100	100	78.1–100	95.7	88.8–98.6

Case definition: animals classified as positive by the DFA model (excluding WB17). Sample used for the calculation of each parameters were: all animals under study (n = 167) for sensitivity, negative reference animals (n = 90) for specificity, cases and negative reference animals (n = 112) for PPV and NPV. Prevalence for PPV and NPV calculation was 19.6%.

## Discussion

This study reports the comparison of 6 different *post-mortem* TB diagnostic methods in naturally infected wild boar. The results show that all diagnostic tests evaluated, performed as described, have limited sensitivity for the detection of *M. bovis*-infected wild boar. Estimated specificity is fair to good for most tests with PCR and gross pathology being the best. In particular, the detection of AFB in histological slides is worthless as a diagnostic technique due to an extremely low sensitivity and will therefore not be discussed further.

Some parameters, like PPV and NPV, are influenced by the prevalence of disease in the population under study. Prevalence in the subset of the sample used to calculate these parameters (16,7%) can be considered typical for TB in free-ranging wild boar populations as those reported are in the range of 2% [5] to 50% [10]. It should be noted that the evaluation of diagnostic methods should be performed under conditions likely to be met in practice [18]. The parameter NPV is especially relevant in the context of game meat inspection schemes, aimed at reducing the risk of human exposure to zoonotic *M. bovis*.

The present results show that wild boar TB survey designs relying exclusively on gross pathology as screening test and culturing only lesion-positive animals significantly underestimate true prevalence. In fact, we estimate that 27.3–27.8% (CI*_P_*_95%_ 10.7–53.6%) of all infected animals might be missed by relying solely on gross pathology as screening test. Similar estimates (25%) were reported for naturally infected white-tailed deer (*Odocoileus virginianus*) that were missed by gross pathology [7]. Moreover, lesions from 2 wild boar, classified as TBL by gross pathology, were found by histopathology to correspond to lesions caused by helminthes and actinogranulomas, in agreement with what has been published elsewhere [8].

The sub-optimal performance of each test under study prompted us to evaluate their combination in parallel. Combination improved sensitivity and NPV, particularly for “stained smears x histopathology I” and “gross pathology x stained smears”. Of particular interest is the highly sensitive combination of “gross pathology x stained smears” (94.4–95.5% sensitivity, 99.3% NPV) as both tests are rapid, cheap and do not require any sophisticated technology. The use of this combination of tests, aimed at selecting animals for bacteriological culture, is therefore strongly recommended in large-scale surveys and game meat inspection schemes.

Bacteriological culture is the reference test for TB diagnosis, as specificity is 100% [1], [8]; nevertheless false negative results do occur [19]. Errors in estimating the sensitivity and specificity of any diagnostic test arise when the reference test does not reach 100% sensitivity and specificity [18] and thus refining the estimated sensitivity of the bacteriological culture for TB in the wild boar is essential.

From our results, DFA classified 5 wild boar with *M. bovis* isolation as negative. The rate of misclassification (4.5%) is extremely low, taking into consideration that bacteriological culture results were not inputted into the model. In 4/5 misclassified animals (note: culture-positive animals classified as negative by the DFA model) no macro or microscopic TBL were detected, but AFB were present. This is consistent with recently infected animals in which lesions did not develop yet [8], suggesting that 18.2% of all infected wild boar in our sample had been recently infected. Another explanation could be that those 5 animals yielded false positive bacteriological results, yet in 1 animal TBL were also detected, supporting the bacteriological classification. In the other 4 wild boar mycobacteria were detected by AFB visualization in tissue smears. Given the precautions taken to avoid cross-contamination between samples, it was improbable that the eventual contamination would be so gross as to allow the detection of AFB in tissue smears performed soon after sample collection.

DFA also classified 4 wild boar without *M. bovis* isolation as infected. Sera from 3 of these animals were submitted to a *post-mortem* serological test [20] and found to have antibodies against *M. bovis*, which further strengthens the assumption that they were infected. All these 4 wild boar presented macro or microscopic TBL and so could be previously infected animals which managed to eliminate *M. bovis* from the organism. Nonetheless in 3 of them we detected mycobacteria, either through visualization of AFB or molecular biology methods. Another possibility is that these lesions contained latent *M. bovis* [21], as reported for *M. tuberculosis* [22]. These results highlight the existence and quantify the probable false negatives associated with bacteriological culture.

Critical factors that can affect the result of bacteriological culture include processing and decontamination of samples, growth media used and the localized nature of mycobacterial distribution in tissues [1], [7]. In this study, tissue samples were frozen for four to seven months before being tested, but Gruft *et al*. have demonstrated that freezing *M. bovis* at −20°C for up to 1 year does not affect its viability [23]. Hexa-decyl-pyridinium chloride has been shown to be the best decontaminant for *M. bovis* isolation, although it decreases *M. bovis* viability at the concentrations used in the present study [24]. The rationale for the high concentration used in the present study is the severe contamination of some of the tissue samples collected, due to the constraints of field collection of samples. Coletsos is an egg-based medium similar to Lowenstein-Jensen with pyruvate, which is widely used for the isolation *M. bovis* (e.g., [5], [7], [11]). We could have failed to detect some infected animals by missing actively infected tissues, especially since, for some animals, incomplete sets of tissues were available for testing. However, this protocol allowed the isolation of *M. bovis* from 5 animals with no detectable macroscopic TBL, suggesting that missing actively infected tissues did not occur at a considerable extent. We should also consider that results from the DFA model are concordant with our bacteriological data.

Although a few reports estimate the sensitivity and specificity of gross pathology compared to bacteriological culture for the diagnosis of TB in the wild boar, there is no comprehensive published evaluation of the different *post-mortem* tests. Martín-Hernando *et al*. reported 82.7% sensitivity for gross pathology [13] and Zanella *et al*. 76.9% sensitivity for the same test [25], both values slightly higher but within the confidence interval of the one we present here. In other wild ungulate species, Fitzgerald *et al*. evaluated histopathology (98% sensitivity, 87% specificity) and detection of AFB (90% sensitivity, 97% specificity) compared to culture in the white-tailed deer [26]. In the same species, 75% sensitivity and 100% specificity for gross pathology have been estimated by O'Brien *et al*. [7]. For red deer (*Cervus elaphus*), Rohonczy *et al*. compared gross pathology (93% sensitivity, 89% specificity), histopathology (88% sensitivity, 89% specificity) and gross pathology x histopathology in parallel (94% sensitivity, 82% specificity) with culture [27]. These results show trends similar to ours in that gross pathology underestimates TB true prevalence and the combination of tests in parallel improves the diagnostic performance.

Our results show the detection of microscopic TBL to be a useful tool in supporting gross pathology suspects in wild boar. In fact, in 16 animals, gross pathology and histopathology I results are concordant; 6 animals were positive for histopathology I and negative for gross pathology; in 2 animals gross pathology lesions were found to be actinogranuloma and parasitic granuloma. Although it does not allow distinguishing TB lesions from those caused by other mycobacteria, as highlighted by the slight comparatively lower specificity of this test [1], histopathology yielded the highest sensitivity of all tests other than bacteriological culture.

The detection of AFB in stained smears correctly identified only over half of the *M. bovis*-infected animals, although estimated specificity was surprisingly high (94.4%). This technique performs better when tissues contain many mycobacteria, which seems not to be the case for most wild boar samples. Though it does not allow the distinction of *M. bovis* from other AFB, it detected only 1/8 MAC and 1/15 other mycobacteria. This test could be a simple and inexpensive technique to strengthen a presumptive diagnosis of TB based on gross pathology.

PCR has the potential for sensitive, specific and rapid diagnosis of TB, but reported sensitivities are well bellow 100% [1], [8]. Molecular biology tests perform worse in tissues containing few mycobacteria, which seems to be the case for most wild boar samples. This may be because of the difficulty of amplifying mycobacterial DNA from samples containing much larger quantities of eukaryotic DNA. [19]. The MPB70 gene has been widely used as a target for the detection of MTC DNA (e.g., [28], [29]). Since DNA extraction is a critical procedure in TB molecular diagnosis, we used bead beating, which was shown to be an efficient technique for extracting mycobacterial DNA from tissue samples [30].

Summarizing, we evaluated for the first time 6 different *post-mortem* TB diagnostic tests in naturally infected free-ranging wild boar. We found that TB surveys based exclusively on gross pathology considerably underestimate prevalence, while combination of tests in parallel improves sensitivity and negative predictive values. Future surveys for TB in the wild boar should use a combination in parallel of gross pathology together with examination of ZN-stained tissue contact smears. All animals positive in any of these tests should be submitted to bacteriological culture for confirmation and molecular epidemiology studies.

TB diagnostic test performance can vary between host species, so these conclusions may only apply to the wild boar. More studies are needed to compare diagnostic tests in other wildlife species, so that epidemiological surveys can be adequately designed as to provide robust data. This is most important where wildlife TB control is being carried out or considered. We have also quantified the probable false negatives of bacteriological culture, which is currently the reference test for TB diagnosis. The occurrence of culture false negatives should be considered when interpreting survey data.

## Supporting Information

Table S1 Diagnostic test results for sampled wild boars with at least one positive diagnostic test result.
(0.13 MB DOC)
