# Supplementary material for: Diagnosis of Tuberculosis in the Wild Boar (Sus scrofa): A Comparison of Methods Applicable to Hunter-Harvested Animals
Source: PLoS One. 2010 Sep 10;5(9):e12663. doi: 10.1371/journal.pone.0012663 (PMC2937024; doi:10.1371/journal.pone.0012663)
Supplement: Table S1 — Diagnostic test results for sampled wild boars with at least one positive diagnostic test result. (0.13 MB DOC) [file pone.0012663.s001.doc]

**Table S1. Diagnostic test results for sampled wild boars with at least one positive diagnostic test result.**

| ID | Study area TB status | Gross pathology | ZN-Stained smear | Histopathology  I | Histopathology  II | PCR MPB70 | Bacteriological  culture |
| --- | --- | --- | --- | --- | --- | --- | --- |
| WB12 | TB | + | - | + | + | + | *M. bovis* |
| WB13 | TB | - | - | + | - | + | *M. bovis* |
| WB15 | TB | - | + | + | - | + |  |
| WB16 | TB | - | - | - | - | - | other mycobacteria |
| WB17 | TB | + | - | - | - | - | other mycobacteria |
| WB18 | TB | + | + | + | - | + | *M. bovis* |
| WB23 | TB | - | - | - | - | - | other mycobacteria |
| WB21 | TB | - | - | - | - | + |  |
| WB35 | TB-free | - | - | - | - | - | other mycobacteria |
| WB39 | TB-free | - | - | + | - | - |  |
| WB41 | TB-free | - | - | + | - | - |  |
| WB43 | TB-free | - | - | + | - | - |  |
| WB46 | TB-free | - | - | - | - | - | other mycobacteria |
| WB47 | TB | + | + | + | - | + | *M. bovis* |
| WB49 | TB | + | - | + | - | - | *M. bovis* |
| WB52 | TB | + | - | + | - | + | *M. bovis* |
| WB54 | TB | + | - | + | - | + | *M. bovis* |
| WB76 | TB | - | - | + | - | - |  |
| WB77 | TB | - | - | - | - | - | other mycobacteria |
| WB79 | TB | + | - | - | + | - |  |
| WB80 | TB | + | + | + | + | + | *M. bovis* |
| WB82 | TB | - | - | - | - | - | *M. avium* |
| WB90 | TB | + | - | + | - | + | *M. bovis* |
| WB91 | TB | + | - | + | - | + | *M. bovis* |
| WB92 | TB | + | + | + | - | + | *M. bovis* |
| WB94 | TB-free | - | - | - | - | - | other mycobacteria |
| WB97 | TB | + | - | + | - | - | *M. bovis* |
| WB114 | TB | - | - | - | - | - | *M. avium* |
| WB117 | TB | + | + | + | + | + | *M. bovis* |
| WB120 | TB | + | + | + | - | - | other mycobacteria |
| WB121 | TB | - | - | - | - | - | other mycobacteria |
| WB123 | TB | - | - | - | - | - | *M. avium* |
| WB124 | TB | - | + | - | - | - | *M. avium* |
| WB125 | TB | - | + | - | - | - |  |
| WB126 | TB | - | - | - | - | - | *M. avium* |
| WB127 | TB | - | + | - | - | - | *M. bovis* |
| WB128 | TB | - | + | - | - | - | *M. bovis* |
| WB130 | TB | - | + | - | - | - | *M. bovis* |
| WB132 | TB-free | - | - | - | - | - | other mycobacteria |
| WB140 | TB-free | - | - | - | - | - | other mycobacteria |
| WB142 | TB-free | - | + | - | - | - |  |
| WB153 | TB-free | - | - | - | - | - | other mycobacteria |
| WB154 | TB-free | - | - | - | - | - | other mycobacteria |
| WB158 | TB-free | - | - | - | - | - | other mycobacteria |
| WB159 | TB-free | - | - | - | - | - | other mycobacteria |
| WB170 | TB | + | - | + | - | + |  |
| WB171 | TB | - | + | - | - | - | *M. bovis* |
| WB172 | TB | + | - | + | - | - |  |
| WB173 | TB | + | + | + | - | + | *M. bovis* |
| WB179 | TB-free | - | + | - | - | - |  |
| WB182 | TB-free | - | + | - | - | - |  |
| WB183 | TB-free | - | - | - | - | - | *M. avium* |
| WB185 | TB-free | - | + | - | - | - |  |
| WB189 | TB-free | - | - | - | - | - | *M. avium* |
| WB191 | TB-free | - | - | - | - | - | *M. avium* |
| WB230 | TB-free | - | + | - | - | - |  |

“-“ negative test result

“+” positive test result

“other mycobacteria” mycobacteria not belonging to MTC or MAC
